# Methods and perceptions of success for patient recruitment in decentralized clinical studies

**DOI:** 10.1017/cts.2023.643

**Published:** 2023-10-06

**Authors:** Brian L. Miyata, Barbara Tafuto, Nadina Jose

**Affiliations:** 1 New Jersey Alliance for Clinical and Translational Science, Newark, NJ, USA; 2 Rutgers School of Health Professions, Rutgers The State University of New Jersey, New Brunswick, NJ, USA

**Keywords:** Decentralized clinical trial, telemedicine, recruitment, retention, diversity

## Abstract

Patient recruitment, diversity, and retention continue to impede successful and representative clinical studies. This systematic review aims to assess the impact of decentralized methods on recruitment, retention, and diversity in recent clinical studies. A systematic search of literature reporting on recruitment in decentralized clinical studies was performed. Studies were reviewed for those reporting the primary outcome of recruitment in decentralized clinical trials, observational studies, or those covering the topic of clinical trials. Secondary outcomes included retention, participant diversity, and participant satisfaction. This systematic search returned 13 studies highlighting the role of decentralized methods impacting participant recruitment, retention, and diversity in clinical studies. Eleven reported improved recruitment using decentralized methods. Seven of these reported improvements directly compared to traditional methods. Seven studies reported positive retention outcomes, with four directly comparing decentralized methods with traditional methods. Six studies were reported to have trended toward increased diversity in the demographics of the sample population, including race or geographic location. Related reviews have stated a lack of published comparable data to determine if decentralized clinical methods improved recruitment and retention. Results suggest this review addresses such a gap, providing data on how decentralized methods such as virtual visits can positively impact recruitment and retention.

## Introduction

Recruitment and retention are leading barriers to the success of clinical trials that have been present even prior to the COVID-19 pandemic. Patient recruitment has been found to be the single biggest cause of clinical trial delays [1]. Up to 20% of clinical trials either face early termination due to failure to recruit or continue to completion while having failed to meet the original recruitment target [2]. Challenges that impact recruitment to clinical trials include issues related to study design, physician attitude, participant attitude, accessibility, demographics, and socioeconomic disparities [3]. The methods and logistics through which clinical studies are conducted have traditionally placed the clinic or hospital at the core. Such a model is referred to as site-centric [4]. Decentralized methods are those that focus clinical study conduct around the patient, using methods such as telemedicine, electronic consent (eConsent), wearable biomarkers, and home visits. Decentralized methods of recruitment include the use of virtual strategies such as social media apps, online campaign advertising targeting specific patient populations, and email blasts to patient databases to improve recruitment and enrollment [5]. Retention is another issue that clinical investigators face, as trials can have up to 40% of the participants dropout [1]. Virtual strategies to improve retention include patient alerts and reminders for visits or completion of questionnaires related to their participation in a clinical study. Varied virtual strategies can not only help ensure accrual but also improve retention, completion, and compliance to the protocol.

Decentralized methods have been incorporated to also improve the diversity represented by the US population in clinical trials. The US Food and Drug Administration (FDA) has drafted guidance regarding how investigators should aim to improve the enrollment of these populations in clinical trials [6]. Offering digital alternatives to increase accessibility to groups who might otherwise have been unable to participate allows for these groups to participate in clinical trials in a more equitable and inclusive manner [7]. In addition to these initiatives focused on increasing diversity, the FDA has issued other guidances acknowledging the potential of leveraging new technologies to improve data acquisition [8].

All aspects of clinical research were disrupted by the onset of the COVID-19 pandemic, suspending ongoing trials, delaying the start of new studies, and hindering participant enrollment [9]. The technology for successful incorporation of digital methods into clinical studies has existed for over 20 years, and although decentralized methods have been used in prior clinical studies, the circumstances brought about by the COVID-19 pandemic created conditions for which their adoption became necessary [10]. In order to mitigate the risks of COVID-19 while resuming research activity, the FDA as well as the National Cancer Institute issued guidance providing flexibility to clinical investigators for the adoption of decentralized methods in conducting clinical trials [11,12]. These guidances suggest the use of virtual clinic visits, delivery of investigational tools to the participant’s home, and use of alternative laboratories or imaging centers to conduct the collection of trial data outside of the traditional, single-site model requiring in-person visits [11,12]. Social media platforms such as Facebook can improve patient recruitment to include rural and other populations typically not represented in a traditional model of clinical studies [13]. Third-party allied health providers such as nurses, physical therapists, phlebotomists, and physician assistants can do home visits to provide protocol-related services [14]. The use of telemedicine and eConsent further increases accessibility for clinical study participants. Since the COVID-19 pandemic, national pharmacies like Walgreens and CVS have set up walk-in clinics where patients can be seen by nurse practitioners and physician assistants [15]. The use of these decentralized methods also allows for more widespread access to patients and enables them to participate in clinical studies in an otherwise nontraditional manner.

Although many trials have now adopted guidance from the FDA and have incorporated decentralized methods into clinical studies, there is still a lack of directly comparable data on how these studies have performed with regard to the aspects of recruitment and retention [16]. The objective of this review is to address this gap in knowledge by conducting a systematic review of the literature and identifying papers that provide insight on how decentralized methods have impacted recruitment, retention, and participant diversity in clinical studies.

## Methods

The objective of this review is to identify peer-reviewed publications reporting on the use of decentralized methods within clinical studies. The following research question was used for primary analysis: How has the implementation of decentralized clinical studies (DCSs) impacted the aspect of recruitment in clinical and translational research?

### Search Strategy and Screening

A systematic search of the literature was conducted to identify relevant articles for the use of decentralized methods in clinical studies and their impact on the aspect of recruitment. As subcategories, this review identified articles that covered aspects of retention and participant diversity in addition to recruitment. A preliminary search for “decentralized clinical trials” (DCTs) was conducted in PubMed to identify interchangeable terms and acronyms to eventually be used in the final search equation. Pearl growing methods were used to identify additional search terms for the search strategy.

Articles were identified by searching in databases PubMed, Cochrane, and EMBASE using a variety of keywords and medical subject headings related to the research question. To ensure that a sufficient number of articles were identified, these keywords included terms pertaining to both clinical trials and observational studies. This was also done by having the complete search syntax included truncated terms to maximize potential article identification. Search terms were limited to include only in title and abstract settings for more precise results. The final search was conducted on October 9, 2022, as follows:

1. Decentral* clinical trial* OR direct-to-participant trial* OR virtual stud* OR virtual trial*
2. enroll* OR recruit*
3. 1 AND 2

**Figure 1.**
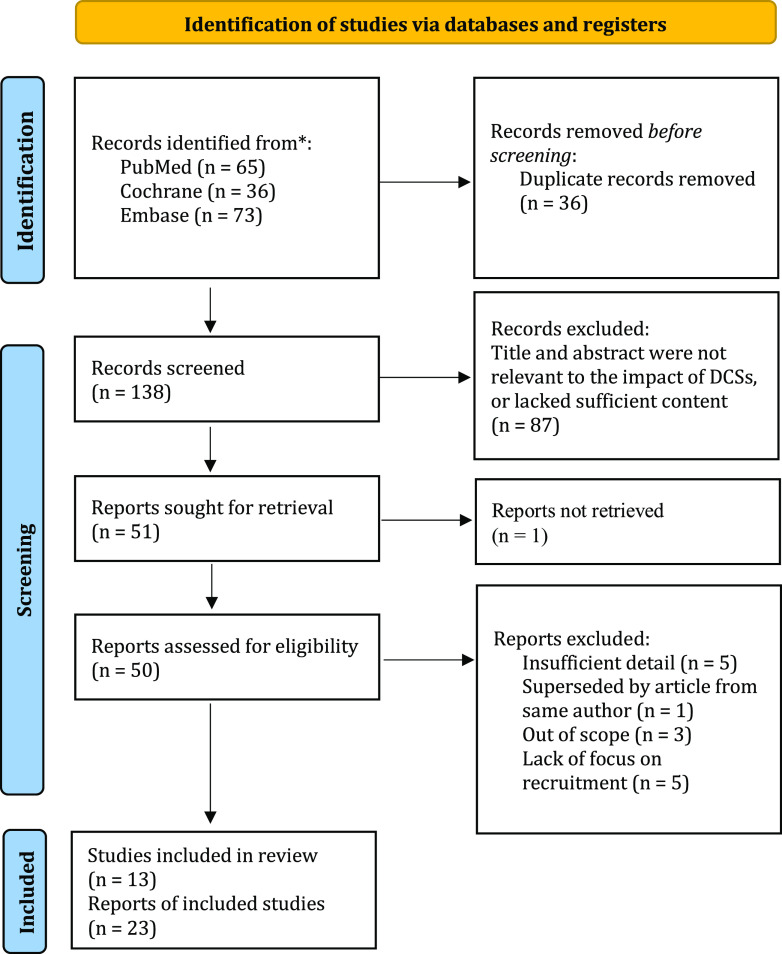
PRISMA flow diagram.

All the identified articles from the initial database searches were exported to EndNote 20 reference management software. Duplicate studies were removed. The titles and abstracts of the articles were reviewed. Relevant articles were retained for eligibility screening. Full-text articles were requested for the remaining articles. Reports were then screened in accordance with the exclusion and inclusion criteria that were set. Studies that included an outcome related to recruitment were included in the final review. The Preferred Reporting Items for Systematic Reviews and Meta-Analyses (PRISMA) statement was used as a guideline. A PRISMA flow diagram depicting the search process used is illustrated in Fig. 1.

### Eligibility Criteria

Following the results of the search conducted in databases PubMed, EMBASE, and Cochrane, articles commenting on the impact of decentralized methods on the aspects of recruitment, retention, and diversity in clinical studies were identified. For the focused review, (1) articles discussing the aspect of participant recruitment in clinical studies implementing decentralized elements were included. Studies including discussion on participant retention and diversity in addition to recruitment were also noted. Other inclusion criteria included the following: (2) study was conducted within the past 5 years, (3) study provides sufficient detail or data with regard to the primary outcome of this review, and (4), study was published in English.

With regard to exclusion criteria, (1) studies conducted prior to 5 years ago were excluded. A time restraint of 5 years was chosen since DCSs largely rely on the current technology available. Therefore, recent articles would provide the most accurate depiction as to how DCSs currently affect clinical and translational research. Other exclusion criteria included the following: (2) studies for which full texts in English were unable to be found, (3) studies that provided insufficient detail with regard to how the study was conducted with decentralized methods, (4) studies that were eventually superseded by another study conducted by the same primary author, and (5) studies that did not focus on the impact of decentralized methods in the context of clinical research.

### Data Extraction

The articles for which full texts were obtained were all logged into a spreadsheet using Microsoft Excel. Collected data points included title, authors, publication year, patient population, decentralized methods used, and primary and secondary outcome information.

### Outcomes

Outcomes that were logged and included in this review are as follows: decentralized methods implemented during the trial, sample population, study design, reported recruitment outcomes, reported retention outcomes, reported participant diversity outcomes, and participant satisfaction. The primary outcome of this review is recruitment outcome. The secondary outcomes of this review are participant retention, diversity, and participant satisfaction.

## Results

The searches initially identified 174 articles. Prior to screening, 36 duplicate articles were removed, leaving 138 left for title and abstract screening. Eighty-seven articles were removed due to lack of relevancy to DCSs or the research question. Full texts were attempted to be obtained for 51 remaining articles, which was successful for 50. The 50 articles were entered into a Microsoft Excel spreadsheet for data extraction and eventual analysis. Fourteen articles were further screened and removed in accordance with the exclusion criteria for this systematic review. Twenty-four articles were included in the review for contextual information and 13 studies provided data to assess the end points of this systematic review. The results of this process are described in a PRISMA flow diagram (Fig. 1). Thirteen studies covering decentralized methods in clinical studies were assessed for outcomes pertaining to recruitment, retention, diversity, and participant satisfaction. These studies are identified in Table 1 and summarized in Table 2, including the type of article and decentralized methods implemented. Table 3 lists the outcomes reported by the investigators pertinent to the focus of this systematic review.

**Table 1.** Publications included in analysis

Study	Type	Study description	Relevant outcome
Adams *et al*., 2022 [17]	Observational	Patients with cancer and survivors of cancer responded to a 41-question, cross-sectional, internet-based survey, taking place between July 6 and September 8, 2021.	Recruitment
Ali *et al*., 2020 [18]	Observational	Subjects with atopic dermatitis were recruited online and given two study tasks per week to be completed with online forms and applications. Patients were given eight DNA lifestyle reports to be unlocked weekly as a reward for protocol adherence.	Recruitment, retention, diversity, and satisfaction
Ali *et al*., 2021 [19]	Observational	Subjects with T2DM were recruited either online or through pharmacy to 3 weeks of glucose monitoring via continuous glucose monitoring devices. Hybrid smartwatches were used to monitor heart rate and track activity and sleep patterns.	Recruitment, retention, and satisfaction
Josan *et al*., 2021 [20]	Observational	Patients over the age of 55 years with atrial fibrillation on oral anticoagulants were recruited virtually or by traditional methods. Patients were enrolled in DeTAP, a single-arm, observational study integrating a suite of digital health technologies to create a 100% virtual trial experience.	Recruitment, retention, and satisfaction
Magnani *et al*., 2021 [21]	Interventional	This trial conducted a four-month intervention in rural patients with atrial fibrillation. Subjects were randomized to mobile health interventions to improve chronic disease management. The interventional arm received a conversational agent, while the control arm used a mobile phone with WebMD and an educational session.	Recruitment, retention, and diversity
Myers *et al*., 2022 [22]	Observational	This research report compares recruitment methods and outcomes of three remote, decentralized observational Parkinson’s disease studies with video visits.	Recruitment, diversity, and satisfaction
Ng *et al*., 2021 [23]	Interventional	The safety and efficacy of intestinal adsorbents in diarrhea-predominant IBS were tested in RELIEVE irritable bowel syndrome with diarrhea (IBS-D), a commercially sponsored, double-blind, placebo-controlled, multicenter study. This study was delivered partly as a traditional site-dependent trial as well as a virtual trial.	Recruitment and retention
Sarraju *et al*., 2022 [24]	Observational	Patients over the age of 55 years with atrial fibrillation on oral anticoagulants were recruited virtually or by traditional methods. Patients were enrolled in DeTAP, a single-arm, observational study integrating a suite of digital health technologies to create a 100% virtual trial experience.	Recruitment, diversity, and satisfaction
Sedhai *et al*., 2022 [25]	Interventional	REDHART2 is a phase II clinical trial in patients with recently decompensated heart failure randomized to receive either anakinra or placebo to improve aerobic exercise capacity. This study was activated at a rural satellite hospital partnered with an urban academic center.	Recruitment and diversity
Sedhai *et al*., 2021 [26]	Interventional	CAN-COVID is a phase III, multicenter, randomized, double-blind, placebo-controlled study enrolling patients with COVID-19-induced pneumonia and cytokine release syndrome (CRS) to assess the safety and efficacy of canakinumab. This study was activated at a rural satellite hospital partnered with an urban academic center.	Recruitment and diversity
Slomovitz *et al*., 2021 [27]	Observational	In this report, accrual data was for Gynecologic Oncology Group (GOG) Partners’ trials over the past four years (2017–July 2020) were collected and analyzed. This information included region of country, institution, and accrual numbers.	Recruitment
Sommer *et al*., 2018 [28]	Observational	This noninterventional study for patients with lower back pain was conducted to compare three different models of clinical trials. The decentralized model had all study visits conducted virtually as well as the consent process. The conventional model had all visits conducted on the investigational site apart from one phone call. Patients could opt for either method in the mixed model.	Recruitment, retention, diversity, and satisfaction
Yiannakou *et al*., 2022 [29]	Interventional	Patients received either intestinal adsorbent Enterosgel or placebo in a randomized, double-blind, placebo-controlled trial for patients aged 16–75 years with IBS-D. Subjects recorded symptoms in a daily eDiary and completed questionnaires. This study adapted to a single-site virtual trial and implemented remote methods in response to the COVID-19 pandemic.	Recruitment and retention

**Table 2.** Description of decentralized methods used and study outcomes

Study	Decentralized method	Intervention and comparator	Primary outcome	Secondary outcome
Adams *et al*., 2022 [17]	Health applications, at-home drug administration, wearable biomarkers, eConsent, and smartphone application	Use of decentralized tools to reduce travel and time for clinical trial participation	Patient likelihood to enroll in cancer trials	
Ali *et al*., 2020 [18]	Siteless, online recruitment, ePRO via smartphone application, and eConsent	Use of patient-centric, siteless, reward-based, and remote trial of patients with atopic dermatitis	Success with nationwide recruitment, identifying patients with variable disease severity, adherence, and dropout	
Ali *et al*., 2021 [19]	Online recruitment, eConsent, online video calls, ePRO via smartphone application, and wearable biomarkers	Use of DCT design elements	Ability to recruit, enroll, and engage patients	
Josan *et al*., 2021 [20]	Online recruitment, eConsent, ePRO via smartphone application, online visits, and study-supplied BP cuff and EKG sensors	Integration of decentralized technologies into a virtual trial experience for patients	Ability of a large phase 3 cardiovascular DCT to achieve quality results and withstand health crises	
Magnani *et al*., 2021 [21]	Smartphone application, telephone-based consent, ePRO, mail-based delivery of assessments, and virtual recruitment	Telephone-based orientation and verbal consent obtainment. Patients were randomized to either receive a smartphone with intervention or control group applications. Study assessments were sent by mail with telephone-based administration and contact for trial duration.	Successful adaptation to virtual engagement and recruitment	
Myers *et al*., 2022 [22]	Online recruitment, digital biomarkers, virtual visits, and ePRO via smartphone application	Remote decentralized observational Parkinson's Disease (PD) studies with video visits	Recruitment processes and outcomes	
Ng *et al*., 2021 [23]	Virtual recruitment and remote virtual trial	Double-blind, placebo-controlled, multicenter study was conducted using both a virtual site and a traditional site	Recruitment efficacy and dropout	
Sarraju *et al*., 2022 [24]	Online recruitment, eConsent, ePRO via smartphone application, online visits, and study-supplied BP cuff and EKG sensors	Integrating digital health technologies into a decentralized clinical trial	Feasibility and success with recruitment, protocol adherence, and engagement	
Sedhai *et al*., 2022 [25]	Telemedicine, remote monitoring, and virtual recruitment	Use of telemedicine, remote monitoring, and institutional oversight to conduct initial steps involved in a clinical trial at a rural satellite hospital	Success with screening, consenting, and enrolling subjects	
Sedhai *et al*., 2021 [26]	Remote monitoring and virtual recruitment	Activating through remote monitoring a multicenter clinical trial at a rural satellite hospital		
Slomovitz *et al*., 2021 [27]	Virtual visits, ePRO, and shipping of drug to patients	Incorporating FDA, CDC, and NIH guidelines, implementing remote monitoring and other remote activities, enhancing enrollment opportunities, and increased frequency of meetings with industry	Describe how the pandemic affected accrual to GOG Partners’ trials	
Sommer *et al*., 2018 [28]	Direct data capture, eConsent, ePRO, wearable biomarkers, virtual visits, and virtual recruitment	Conducting trial with decentralized, conventional, and mixed model clinical trial settings	Comparing decentralized, conventional, and mixed models for conducting a clinical trial	Comparing operational and recruitment methodology of the models, assessing participant satisfaction, physical activity, and body posture, and evaluating patient compliance with reporting via eDiary
Yiannakou *et al*., 2022 [29]	Virtual visits, ePRO, eConsent, and virtual recruitment	Enterosgel for the treatment of IBS-D with diarrhea compared with placebo	Percentage of responders in the treatment group compared to placebo group	

**Table 3.** Primary and secondary outcomes of systematic review

Study	Recruitment outcome	Retention outcome	Diversity outcome	Participant satisfaction
Adams *et al*., 2022 [17]	Self-reported increase in likelihood to enroll in decentralized trials reducing need for travel			
Ali *et al*., 2020 [18]	Rapid recruitment rate of 55 in less than 1 month using Facebook compared with median recruitment in UK of 9.2 subjects per site per month.	Authors report high retention rate of 96% due to ease of participation	Majority of participants were from rural areas with low physician coverage. Facebook recruitment may limit ability to recruit broadly since the study mainly recruited young women.	90% of participants reported enthusiasm for future or longer study
Ali *et al*., 2021 [19]	Online recruitment found to be superior to traditional recruitment in cost-effectiveness and time efficiency	Retention rate of 87%		85% preferred online informed consent conversation
Josan *et al*., 2021 [20]	Dramatic surge in enrollment compared to traditional methods upon implementation of social media advertisements	High retention rate reported		90% reported strong interest in future DCT participation
Magnani *et al*., 2021 [21]	Authors state strengths of virtual recruitment are evident, enrolling 130 individuals after adapting from traditional methods	92% retention of participants enrolled with virtual methods	Remote and rural individuals were recruited	
Myers *et al*., 2022 [22]	All studies completed enrollment, with only five reaching its prespecified goal. Two out of three studies reported increased recruitment after modifying recruitment process		Geographic distribution of patients spanned 45 states, with 30.3–42.9% of participants coming from areas lacking primary care	Participants across all three studies report high satisfaction with video visits
Ng *et al*., 2021 [23]	Virtual recruitment found to significantly increase recruitment rates	Virtual trial demonstrated improved retention compared to site-dependent trial		
Sarraju *et al*., 2022 [24]	Social media recruitment implementation resulted in dramatic recruitment surge		Adjustments should be made to achieve racial and ethnic diversity, since majority of participants were White and urban-dwelling	86% of survey respondents expressed willingness for future DCT participation
Sedhai *et al*., 2022 [25]	Recruitment in progress with target of 102. Authors report accelerated enrollment. Sixty-one enrolled from January 2019 to August 2021, nine of which were enrolled from partner community hospital.		Study anticipates enrolling 50% of subjects who are historically underrepresented minorities	
Sedhai *et al*., 2021 [26]	Study surpassed enrollment goal of 40, enrolling 51 subjects from May 8, 2020 to August 13, 2020, 16 of which were enrolled from partner community hospital.		Study anticipates enrolling 40% of subjects who are historically underrepresented minorities	
Slomovitz *et al*., 2021 [27]	Authors report accrual to GOG Partners’ trials increased 37% over the median monthly accrual since the pandemic began			
Sommer *et al*., 2018 [28]	Higher recruitment rate was observed in decentralized arm compared with conventional arm	Higher retention observed in decentralized arm (89%) than conventional (60%)	Telemedicine center was able to recruit more broadly compared with health clinic	Patients generally satisfied with eConsent, eDiary, and remote visits. Lower satisfaction voiced with patch sensors used
Yiannakou *et al*., 2022 [29]	Virtual recruitment was significantly faster than total recruitment from traditional sites. 440 patients were randomized, 270 via traditional and 170 via virtual sites.	Retention rate was significantly better from virtual sites compared to traditional.		

The articles identified (Table 1) provide detailed data and insight from investigators as to how decentralization has impacted clinical studies. Here, the decentralized methods commonly used are outlined by study (Table 2). Ten of the 13 articles contain information directly from completed or ongoing studies, while 3 articles are surveys or reports evaluating the impact of recruitment on clinical trials. Eight of the 13 studies have been conducted in the USA, while the other studies have been solely conducted in European countries.

### Decentralized Methods

The studies identified in this review incorporated methods that leverage technology or other means to deviate from the traditional, single-site model historically employed to conduct clinical trials. Common methods mentioned among these 13 studies include virtual recruitment, electronic patient reported outcomes (ePRO) through wearable biomarkers or smartphone applications, eConsent forms, and virtual visits.

### Recruitment

Eleven out of 13 studies report recruitment success implementing social media advertisements or other methods deviating from the traditional approach where all recruitment is conducted in the clinic. In these studies, success was declared through the achieved recruitment rate, the number of participants enrolled, or the ability to meet the recruitment goals of the study. Of the two other studies, one was a survey in which participants expressed likelihood to enroll in trials using decentralized methods to lessen travel burden. The other study only voiced improved recruitment in two of the three trials covered. This review identified studies that directly compared virtual recruitment strategies alongside traditional methods. Seven studies made direct comparisons between the two, where outcomes such as time to reach recruitment goals and number of enrolled participants favored virtual methods instead of in-clinic, traditional methods of recruitment. When commenting about the benefits of virtual recruitment, authors often noted faster recruitment rates and cost-effectiveness, with some studies having to place potential subjects on waitlists due to the dramatic surge in recruitment that was observed.

### Retention

Seven out of 13 studies were found to report success with retaining subjects in studies implementing decentralized methods, potentially attributing the study’s success to the ease of participation for subjects. Four studies included direct comparisons between retention in DCSs with traditional methods. These comparisons often occurred by comparing retention of participants recruited prior to the pandemic with retention of participants recruited after the pandemic forced trials to adopt virtual methods. In other articles, decentralized and traditional arms occurred simultaneously in the study, or retention in the decentralized study was compared with the standard retention of other studies for that time.

### Diversity

Six out of 13 studies reported some benefits to sample population diversity in DCSs. Diversity was usually observed in the ability to recruit geographically remote subjects, often from rural areas lacking adequate healthcare coverage. Two studies conducting DCSs noted potential limitations in their ability to reach certain populations, with those investigators suggesting that adjustments should be made to reach historically underrepresented racial and ethnic groups. Two other studies set specific goals to reach underrepresented minorities. These studies involved partnering a community hospital with a larger academic center to conduct clinical trials employing decentralized methods, and both studies were successful in achieving their goals pertaining to participant diversity. The authors attribute this success with diversity to the use of telemedicine and uniform access to electronic health records (EHRs) across sites, which they used to overcome geographical boundaries and attract diverse participants representative of the target populations.

### Satisfaction

Six studies reported on participant satisfaction, all of which included positive feedback. Satisfaction was expressed in a variety of ways, such as enthusiasm for participation in future DCSs or preference toward the decentralized methods implemented. Participant satisfaction was often measured through feedback questionnaires that took place either during the study or after completion of the study. One study noted high satisfaction with the informed consent process being conducted virtually. Three studies included enthusiasm for participation in future DCSs.

## Discussion

The goal of this systematic review was to identify how the implementation of decentralized methods has impacted the ability of clinical studies to overcome long-standing barriers to success such as participant recruitment. The identified papers covered clinical trials and studies that were conducted using methods such as wearable biomarkers, eConsent, and virtual visits, to provide accessibility that may not be present in the traditional, single-site study. This review provides data from studies on the use of these decentralized methods for recruitment, retention, diversity, and participant satisfaction. Overall, the findings of this systematic review provide further support in favor of implementing decentralized methods in clinical studies. The results are consistent with prior literature identifying the advantages associated with decentralized methods [16,30].

Previous systematic reviews conducted to assess the impact of decentralization on clinical trials have identified the advantages associated with these methods [16,30]. However, these reviews have stated that there is still a lack of definitive support for whether decentralized methods are beneficial for recruitment, retention, adherence, and cost metrics [16]. This may result in hesitation from clinical investigators to adopt these methods in future trials. This review provides data from studies, many of which started implementing decentralized methods as a result of the COVID-19 pandemic, on the aspects of recruitment, retention, diversity, and participant satisfaction.

Participant recruitment is one of the largest barriers to successful clinical trials, and the included articles demonstrate that investigators are willing to implement decentralized methods to access potential participants through nontraditional means [1]. The studies identified in this review reported faster recruitment rates and higher amounts of enrolled participants through conducting the enrollment process virtually or by using social media advertisements [18–29]. The strongest evidence comes from the trials that have conducted recruitment through virtual and traditional methods, comparing the outcomes between the two [18–21,23,24,28,29]. In addition, presenting patients with the opportunity to enroll in clinical trials implementing decentralized methods has also been shown to increase self-reported likelihood to enroll [17]. As investigators continue to evaluate the benefit to recruitment that DCSs have, it should be noted that parameters such as conversion rate should be considered differently. In virtual recruitment, conversion rate is calculated by dividing the number of individuals signing up for the study by the number of individuals who visited the recruitment website and clicked on the button to participate [18]. Although conversion rates with virtual recruitment may be lower compared to conversion rates through conventional recruitment, online recruitment reaches a higher number of people and conversion rates for virtual studies above 5% are considered good [18].

Though conducting the initial search for this review focused on participant recruitment in DCSs, it was noted that many articles also commented on how decentralized methods impacted other elements such as retention. Secondary outcomes evaluated in this review included retention, diversity, and participant satisfaction in studies utilizing decentralized methods. Articles reporting on these outcomes in clinical studies have largely been in favor of decentralized methods, reporting positive outcomes such as high retention rates, better inclusion of historically underrepresented populations, and enthusiasm for future participation in DCSs. In order to reduce health disparities in the USA, increase the generalizability of results, and promote equity in healthcare, clinical investigators have an ethical duty to take actionable steps such as leveraging digital tools to decrease the burden of participation for underserved populations. Through implementing telemedicine into clinical trials, geographical boundaries are nearly eliminated for potential participants that may have been previously unable to access trials. DCSs operating through community hospitals partnered with larger academic centers have shown success in reaching enrollment goals focused on participant diversity. The studies assessing this model utilized a satellite hospital serving a geographical area designated as a healthcare shortage area by the Health Resources and Service Administration. This hospital, which serves rural and underserved populations, received institutional oversight from a larger academic medical center. One way this lead institution provided oversight included having the principal investigators and main research coordinators located there and providing training for the other physician investigators located at the community hospital via videoconferencing [25,26]. Increasing the ease of participation in clinical studies has demonstrated a positive impact on patient retention in DCSs [18]. Many of these studies had participants complete study-related tasks from their homes through eDiaries or mobile applications. One study implementing a rewards-based system for task completion had more than 50% of the participants report increased motivation to progress with the trial due to this design [18]. Similar methods of engagement such as sending newsletters to patients, easily consumable videos, or providing patients with appropriate data relevant to the trial may show similar success. Participants in DCTs also expressed interest in future clinical trial participation where decentralized methods are being utilized [24].

Implementing decentralized methods promotes study design and conduct that moves away from that of the traditional, single-site model. The use of satellite hospitals is one of many examples of the potential for new models of trial oversight. The use of decentralized methods in clinical trials was gaining support from regulatory agencies prior to the onset of the COVID-19 pandemic. In December 2018, the FDA announced a new strategic framework to advance how investigators utilize real-world evidence (RWE) in supporting the development of drugs and biologics [4]. This framework acknowledges how the healthcare system is finding more effective methods to leverage electronic tools to gather health-related information during routine care of patients, referred to as real-world data. The FDA has already allowed the use of RWE to eliminate the need for post-marketing studies on nine potential safety issues in five products, and the framework includes efforts to utilize RWE to help support the approval of new indications for approved drugs [8]. Following the onset of the pandemic, the advantages of a patient-centric model of conducting clinical trials over the traditional, site-centric model are becoming clearer. In response to the rapid adoption of decentralized methods by investigators, the FDA is requesting the applicants of new drug application/biological license applications (NDA/BLA) to indicate when data points were collected using remote means, looking to determine the potential risks and benefits associated with DCT solutions [4].

Several studies in this review reported limitations with the use of decentralized methods. In one study utilizing social media recruitment through Facebook, the majority of participants enrolled were young women even though the average age of adults using Facebook is 48.2. These investigators have recommended future studies to consider conducting online recruitment through additional channels, such as the websites of patient associations or pharmacies, in order to address this bias in recruitment [18]. Reported outcomes for participant satisfaction are also at risk for bias. High rates of reported satisfaction may be skewed, since questionnaires were often optional and only open to individuals who enrolled in the study, which could indicate precluding interest and enthusiasm in the study.

The results from this systematic review of studies conducted in the last 5 years demonstrated the potential of decentralized methods to enhance the conduct of clinical studies toward a patient-centered model. The inclusion of studies done during the years of the COVID-19 pandemic accelerated the use of these tools as well as strategies to mitigate challenges. Recruitment may be improved in DCSs due to the virtual recruiting methods employed or because of the increased accessibility granted in these trials. The increased accessibility that DCSs demonstrate is often met with participant satisfaction, and therefore, it should be noted that the implementation of patient-centered tools is being positively received. The resulting data from this analysis lessen the gap of documented information needed to justify the integration of decentralized methods when designing clinical studies. Future DCSs should continue to evaluate and report on these methods to better understand the impact on recruitment, retention, diversity, and participant satisfaction.
